# The benefits of working abroad for British General Practice trainee doctors: the London deanery out of programme experience in South Africa

**DOI:** 10.1186/s12909-015-0447-6

**Published:** 2015-10-14

**Authors:** Candice Reardon, Gavin George, Oluwatobi Enigbokan

**Affiliations:** Health Economics and HIV and AIDS Research Division (HEARD), University of KwaZulu-Natal, Private Bag x54001, Durban, 4000 South Africa

**Keywords:** Out of Programme Experience (OOPE), Doctors, South Africa, United Kingdom, International health, Medical schools

## Abstract

**Background:**

The value of international health experience for doctors from developed nations is well recognised. Provisions have been made for medical staff in the United Kingdom to embark on work experiences abroad during their careers in the National Health Service. The London Deanery and Africa Health Placements provide an Out of Programme Experience for British General Practice trainee doctors wanting to work for a year in rural hospitals in South Africa.

**Methods:**

A qualitative study was conducted among fifteen British General Practice trainees who participated in the programme. The research aim was to understand the perceived benefit and value of their experience and their opinions about the structure of the programme. The data was analysed using thematic analysis.

**Results:**

Their experience provided an accelerated year of learning and development that contributed to their professional and personal development. In addition to their general development, their improved ability to work in resource limited settings, enhancement of soft skills, a greater appreciation for the National Health Service and a better understanding of working within foreign health care systems were important gains. The timing of the experience, the security of re-employment on their return, assistance with administrative requirements of destination countries and the opportunity to gain varied, hands-on experience were highly valued components of the Out of Programme Experience.

**Discussion:**

The value and benefits derived from the doctors' experience in South Africa are discussed in relation to another evaluation of the Out of Programme Experience, as well as issues of transferability of skills and competencies and future impacts on career decisions.

**Conclusion:**

This study provides evidence to suggest programmes such as the OOPE have the potential to create substantial benefits for trainee doctors, both in terms of their medical skills and competencies and through the development of softer skills. This programme, through the supply of scarce skills, further benefits the host country and specifically the health facilities and communities served by these trainee doctors.

## Background

Over the past decade, an increased number of United Kingdom (UK) health professionals have sought work experience abroad within foreign health systems [1]. The importance of international experience in health is well recognised by the UK and supported by a number of policies and recommendations. *A reference guide for postgraduate speciality training in the UK: The Gold Guide 2009* [2] makes special provision for taking time out of programme for clinical experience where this clinical experience supports the recommendations made by *Global Health Partnerships: the UK Contribution to Health in Developing Countries (2007)* [3]*.* A seminal report of its time, *Global Health Partnerships (2007)* recognised that strong global partnerships for health are instrumental for helping achieve the Millennium Development Goals and promoting health in developing countries. Several recommendations from *Global Health Partnerships* call for arrangements to be made with organisations to enable National Health Services (NHS) staff to gain valuable work experience abroad that is targeted to the express needs of developing nations without being penalised or disadvantaged, with provisions made for continued employment or re-employment on their return. It is accepted that this international health experience will enhance UK doctors’ understanding and expertise in managing different medical conditions (particularly tropical diseases) whilst working within foreign health systems and resource limited settings [1, 3], ultimately enhancing the development of medical professionals for the NHS.

The introduction of modernising medical careers (MMC) in the United Kingdom in 2005 has also made provision for out of programme work opportunities in developing nations. The MMC serves to accelerate medical training, guaranteeing that the fundamental abilities and skills of doctors are preserved [4]. The programme was established in 2005 to improve the postgraduate medical training system and has made provision for out of programme placement opportunities [5]. Support for these opportunities to work abroad ensures that more doctors are enlisted and equipped with the skills required to alleviate the strain of staff shortages in developing nations [6]. However, there is doubt as to whether existing training programmes suitably prepare UK doctors to make a significant contribution to their host country, especially within foreign health systems grappling with severe resource shortages [1]. Brown and colleagues have called for increased integration of global health training within speciality programmes in the UK that can better prepare doctors for working in developing nations [1].

Despite these concerns about adequate preparation, it is recognised that health students and staff, and their sending countries, can derive considerable benefit from international health opportunities, including improved self-confidence and adaptability [6], awareness of global health concerns [6, 7], increased knowledge and clinical skills for managing less common tropical diseases [6, 8], more severe pathologies [9] and/or those diseases affecting foreign and migrant populations within sending countries [6], better utilisation of resources in resource deprived settings [9], and improved surgical skills [8, 9]. Research also shows that health experience gained abroad in resource limited settings can generate greater compassion toward and willingness to work among disadvantaged populations [7, 8] and may alter medical students’ career plans towards primary health care and public health [8, 10], although long term impacts on career paths have not been adequately established.

### The London deanery out of programme experience in South Africa

In 2008 the London Deanery together with Africa Health Placements (AHP), a social profit organisation based in South Africa (SA), established an Out of Programme Experience (OOPE) for UK trainee General Practitioners (GPs). Trainee GPs spend a year between their second and third year of GP speciality training working at a rural hospital in SA. The programme is voluntary and at the time of the research study did not earn doctors any accreditation towards their GP speciality. The process of arranging for the trainee GPs to come to SA is facilitated by AHP and two co-ordinators; one based in the UK and one in SA. The hospitals to which they are allocated employ the candidates as a Medical Officer for one year; their salary paid for by the South African Department of Health. Costs accompanying the administration process and arrival and departure from SA are borne by the trainee GPs themselves. During their OOPE, the trainee GPs must complete an e-portfolio. This is a continuous learning assessment portfolio which the doctors are required to update frequently with evidence of newly acquired clinical experience and skills.

The OOPE was established with two objectives in mind; firstly to provide a sustainable inflow of British GP trainees to rural health facilities in SA which would in turn alleviate health staff shortages. SA has a substantial shortage of medical doctors in its rural health facilities as a result of both internal migration (from rural to urban areas) and emigration [11]. Actions taken by the South African government to address staff shortages in rural areas include bilateral agreements with countries to mitigate the outflow of SA health professionals or facilitate the inflow of foreign health professionals to rural areas, community service for graduating health professionals, salary increases via the Occupational Specific Dispensation and rural allowances [11]. Effective time-limited placements of foreign HPs in rural areas in SA through OOPE is one such strategy of capacitating rural health facilities.

The second objective is to enhance the professional development of the UK doctors themselves. According to the OOPE Director at the London Deanery, the placements enable GP trainees to enhance their competencies around taking initiative, leadership, dealing with human resource constraints and exposure to global health issues. Experiences and skills are gained in areas that would be difficult to achieve within their existing three year specialty training in the UK [12]. To date, there have been OOPE doctors placed in Uganda, Malawi, SA, Madagascar, Solomon Islands, Northern India, Cook Islands, Nicaragua, Zambia and Costa Rica [13].

This paper adds to previous research undertaken on the OOPE that explored the experiences of these British trainee GPs working in rural hospitals in SA as part of their OOPE, whilst revealing the reasons and motivations underlying their decision to come to SA [14]. This paper explores the perceived benefit and value trainee GPs derived from their OOPE, and their views about the structure and operation of the OOPE.

## Methods

### Design and sample

This paper is based on the findings of a qualitative, exploratory study conducted in 2011. Altogether fifteen UK GP trainee doctors were interviewed. This figure included eleven British doctors who were in SA as part of the OOPE between August 2010 – July 2011 (whom we refer to as ‘current OOPE participants’) and four deanery candidates who had been on the OOPE in 2008 (one participant) and 2009 to 2010 (three participants) (whom we refer to as ‘past OOPE participants’).

### Setting

The participants were currently in or had been stationed at 11 rural hospitals, 10 of these in Kwa-Zulu Natal and one in the Eastern Cape. Ten hospitals were district hospitals and one was a combined district and regional hospital. District hospitals (level 1) play a critical role in supporting primary health care, acting as a gateway to more specialist care. District hospitals provide generalist services to in-patients and outpatients and are serviced by medical doctors who are generalists (ordinary GPs). They can have between 30 and 200 beds, a 24-h emergency service, access to basic diagnostic equipment and an operating theatre. There would be no intensive care unit. Regional hospitals (level 2), conversely, are staffed by specialists and general practitioners and provide speciality services in at least five of the following areas: surgery, medicine, orthopaedics, paediatrics, obstetrics and gynaecology, psychiatry, diagnostic radiology and anaesthetics. They have between 300 and 600 beds and see between 300 and 800 outpatients per day.

### Research instruments

Two interview schedules were used with current and past OOPE participants. The interview schedules were similar, with the one developed for past OOPE participants probing the benefits of the OOPE for trainee doctors after their return to the UK. The interview schedules were semi structured, containing a primary question and several probes in each section that enabled the researcher to gather information about different aspects of the GP trainees’ experience on the OOPE. The main sections in the interview schedules covered: (a) their reasons for embarking on the OOPE, (b) their preparation for the OOPE, (c) their experiences of living and working in SA, (d) their goals and aims for their OOPE, (e) the benefit and value they derived from it and (f) their views and opinions about the structure of the programme. The findings presented in this paper focus on data from the latter two sections.

### Data collection and analysis

Interviews with the doctors were conducted in English and lasted between 30 and 45 min. They were all conducted by the same interviewer – an English speaking, female researcher at the Health Economics and HIV and AIDS Research Division with no relationship to the interviewees. Interviews with those doctors based in the UK (*n* = 4) and one based in the Eastern Cape were conducted telephonically. The remainder (*n* = 11) were conducted personally with the participants at their places of employment. All the interviews were recorded and transcribed verbatim. Verbal and written consent was provided by the interviewees prior to the commencement of the interview.

The researcher who conducted the interviews, the primary author of the paper, was responsible for analysing the data, and did so manually. The same framework used to gather the data was used to guide the data analysis. The data was analysed in accordance with these main themes of the GP trainees’ experience and was guided by the methodology of thematic analysis [15, 16]. The use of a pre-existing framework of overarching themes in research has been referred to as ‘a priori themes’ and is recommended as long as the researchers are open to the emergence of new ideas and codes from the text [15]. Ryan and Bernard’s techniques for identifying themes from basic expressions in the text were used to guide the process by which sub themes were identified from the text [16].

As one researcher was assigned to the analysis of the qualitative findings, inter coder reliability could not be used to verify the consistency of the codes used. Instead, transcription checking and the use of constant comparisons to prevent definitional drift in the application of codes [15] were used to ensure reliability of the findings. The transcriptions were checked twice, in some cases three times, by the individual responsible for the transcription to ensure that the transcripts included no obvious mistakes. The researcher also checked portions of the transcripts to ensure their accuracy. To guard against definitional drift in the coding process, the researcher developed coding definitions for each of the codes to ensure the consistent use of codes across cases and during earlier and later stages of the analysis. The use of constant comparisons during analysis, as discussed above, also assisted in identifying inconsistencies in the application of codes across cases. The researcher also looked for variations in the data, or negative case analysis, which were then examined in more detail to understand why the variation occurred [15].

### Ethics statement

Ethical approval was gained from the University of KwaZulu-Natal (UKZN) Biomedical Research Ethics Committee (Ref: BE191/010) as well as from the Kwa-Zulu- Natal Provincial Department of Health (Ref: HRKM146/10).

## Results

### The value and benefit of the OOPE

Every trainee GP reported having benefited from their OOPE. These narratives were categorised into four themes covering their maturation as doctors, their improved ability to manage scarce resources, a broadened perspective of international health care systems and the enhancement of their softer skills.

#### Maturation as a doctor

Living and working within resource constrained settings played a crucial role in their maturation as doctors. One doctor pointed out that her stressful work environment had been a major catalyst to her development as a doctor:

*“I think just living in a more stressful environment like that, you just mature. It’s like having an accelerated year of learning so it’s almost like that one year in SA felt like the equivalent of what you’d learn in 3 or 4 years in the UK, just because it’s so intense”*. (Current OOPE participant, public hospital, northern KZN)

Four doctors described the benefit and value of the OOPE more generally, believing that it had made them “better doctors in a lot of ways” and “more well-rounded” as GPs. Two doctors felt the opportunity to learn new skills and work in different specialities was helpful in deciding whether they wanted to continue working as a GP or embark on another speciality, as is reflected in the following quote:

*“Career wise it would be a lot better if I were to pick obs and gynae [obstetrics and gynaecology], I’m already so many years ahead of the similar level of trainees back in the UK. Whereas in obs and gynae training you wouldn’t be expected to be competent in caesarean sections until you’re in year three. Whereas I’m quite happy to say that I’ve done about 20–30 caesarean sections here now and I can do it”. (Current OOPE participant, rural hospital, inland KZN)*

A number of doctors highlighted ways in which they believed their experience in SA would benefit the NHS. This included their experience gained in the treatment and management of tuberculosis (TB) (noted to be a rising problem in London), their generalist skills gained through working within various specialities, the conditions and pathologies encountered in SA, and the subsequent enhancement of their clinical judgement and skills. Several doctors credited the OOPE with increasing their confidence to practice medicine. Their increased confidence was, in their opinion, both learned and exhibited in their ability to: manage their own doubt and their patients’ uncertainty; dealing with extraordinary challenges and conditions such as conducting caesarean sections, treating patients with severe HIV related diseases including TB and cryptococcal meningitis; becoming comfortable with working autonomously and independently; and making better decisions for their patients. The following quote shows how one doctors’ improved confidence enabled her to make a timely and ultimately better decision for her patient:

*“…we had a really difficult decision to make…and I said ‘No, no. We really have to transfer her, it’s just not safe’… it was definitely the right choice to transfer her because she would have died at the hospital. I was really surprised that I could be confident enough to make that decision, to stand up for the decision I thought was right.” (Past OOPE participant, public hospital, Eastern Cape)*

#### Improved management of scarce resources

Working within a resource constrained setting was found to have several positive consequences. The majority believed it made them better decision makers when it came to patient care. It reportedly forced some doctors “to think of the bigger picture” and to not only “treat the patient in front of you”. One doctor described herself as having been “quite blind as to what things cost and the limits of things”, whereas after working within a rural hospital for a year she better understood how to allocate and work with scarce resources. Another doctor believed that learning how to work within a resource limited setting had helped her make more considered decisions as a doctor:

*“So when I came back to the UK I was much more sensible with what I was requesting, what tests I was doing. I wasn’t just throwing off all these meticulous crazy tests for no reason. It made me more mature in my decisions about how to manage patients. I knew about rationing and about what was sensible to do and what was not.” (Past OOPE participant, Public hospital, Northern KZN)*

#### Increased perspective of international health care systems

Five trainee GPs mentioned that the OOPE had given them a broader perspective of international health care systems. These doctors agreed it had been valuable to understand how health care systems operated in places very different to the UK. Experience working with foreign populations, according to two doctors, was something that would benefit them working in the NHS in London, because of the increasing number of foreigners, including people from sub-Saharan Africa, accessing NHS services. The experience of working in a different health care system gave four doctors a new appreciation for the NHS and the standard of living they enjoyed in the UK.

#### Enhancement of softer skills

The doctors also reported a number of “soft skills” that their experience in SA helped develop. This resulted from the language difficulties and cultural obstacles between them and their patients, the added responsibility and seniority they held due to staff shortages; the latter requiring effective leadership and teamwork critical to the delivery of health care.

Working closely with health staff ensured that skills in leadership and management, team work, problem solving, negotiation, conflict resolution and diplomacy were nurtured. Leadership and management skills were reportedly honed through doctors’ participation in different hospital committees, as well as their relative seniority in the hospitals. About half the doctors felt they were occupying a more senior role with greater responsibility to what they were used to in the UK. In the following quote, one doctor describes how her senior role in managing teams at her hospital had been one of the most positive experiences of her OOPE:

*“I think the best thing is the development of the doctor because… for the first time you’re actually in a position where you’re properly running teams particularly when you’re on call. I guess leadership skills, in the UK you don’t have that as a junior doctor, you’re always quite wrapped in cotton wool, but here you are running teams.” (Current OOPE participant, rural hospital, northern KZN)*

Language and cultural barriers between the doctors and their patients meant that the doctors’ verbal as well as nonverbal communication skills had to improve. Many had to rely on nonverbal skills to communicate their empathy and support. Three doctors mentioned that working with patients from different cultural groups had helped increase cultural sensitivity and awareness.

### The structure and operation of the OOPE

Most doctors agreed that the timing of the OOPE between their second and third specialist training years was ideal, both in their personal lives and in their training. Two doctors believed that the generalist skills they gained in their first two years of GP training adequately prepared them for SA where they “needed to be as experienced as possible while still being generalists”.

Secondly, almost every doctor made mention of the importance of having a job to come back to in the UK upon their return. The guarantee of a job on their return made the programme more “accessible” to the doctors and more appealing for them to embark on a year out of training.

Having an in-country partner like AHP to assist with the substantial amount of administration was of great comfort to many of the doctors. The doctors relied on AHP’s assistance in facilitating their arrival and helping them to secure posts in SA. This was deemed crucial to “making the transition a lot easier” for the trainee GPs.

With regard to their salaries, without prompting, five doctors reported being pleasantly surprised with the salaries they received in SA, which they felt compared favourably with the UK. Two doctors felt they were able to earn more during their OOPE than they would have if they were in the UK. Several doctors also articulated positive views about the hands-on experience they received in their OOPE that was far more than they would have gained back in the UK.

Apart from the three doctors within the pilot programme who had a portion of their OOPE accredited, the majority did not receive any credits towards their GP training. However, almost every doctor was supportive of this no-accreditation policy. Firstly, some were in no rush to become a GP and they viewed this OOPE as an added experience that would benefit them in their progression to becoming a qualified GP. Secondly, although the OOPE was viewed positively in terms of their development as a doctor, three doctors said that they did not want to have their UK speciality training time reduced because of this experience.

Only one aspect of the programme’s operation elicited complaints and negative views from the doctors. Almost every doctor that was currently involved in their OOPE had experienced some difficulty in adhering to the requirements of their e-portfolio. Difficulties in updating their e-portfolios stemmed from the lack of adequate internet access at their hospitals and staff shortages at their health facilities, which made them reluctant to burden senior doctors with the completion of their e-portfolio requirements.

## Discussion

The findings indicate that the majority of the doctors derived substantial benefit and value from their OOPE. The sense of becoming more well-rounded and maturing as a doctor stemmed in part from the nature of working within district hospitals that exposed doctors to a wider range of medical conditions and required a broader range of knowledge and skills than that associated with working in only one speciality area. The shortage of resources and staff, the severe cases (most commonly HIV-related), and procedures they were performing and their senior role in the hospitals also served to accelerate their learning and development.

A large portion of the trainee GPs’ maturation was attributed to their experience in having to work with resource shortages that forced greater reliance on their clinical skills and judgement. The opportunity to understand how different health care systems operated and their experience gained in working with foreign populations, improving their cultural awareness and sensitivity, were also notable benefits raised by participants. In addition to their clinical experience, the doctors reported that the OOPE had improved their soft skills such as team work, leadership, management and problem solving skills, contributing to their holistic development as doctors. The majority of the doctors welcomed the opportunity to lead teams and to work with more autonomy and responsibility than they were accustomed to in the UK. These findings confirm another evaluation of the OOPE where junior doctors were found to have developed their clinical skills alongside generic skills such as leadership, management and decision-making, as well as better use of resources [17]. This paper does, however, caution that not all clinical skill improvements were directly transferable to the doctor’s clinical work on return to the UK [17]. In our study, clinical skills and competencies to perform more specialised procedures were advanced in the context of less hierarchical structures and the greater autonomy and independence with which the Deanery candidates worked. Their work environment in the UK may not allow for the performance of these new skills and competencies; however gains in confidence and softer skills will be more easily transferable to UK work contexts.

Most doctors expressed favourable views and opinions about the structure and operation of the OOPE, apart from difficulties with maintaining their e-portfolios. The doctors held positive views about the timing of the OOPE between their second and third year of speciality training, the salaries they earned in SA and, at the time of the study, the no accreditation policy of the programme. The guarantee of re-employment in the NHS on their return and the administrative assistance and support given by AHP in SA emerged as critical assets to the programme’s success. The guarantee of a job after returning from international health experiences is well recognised by the UK as an essential element for promoting these experiences [3]. It was also noted that the UK system such as the MMC and European working time directives had substantially reduced the amount of on-the-job experience junior doctors would receive in the NHS, delaying this to later stages in their careers. Therefore, the opportunity to gain experience in treating the variety and severity of medical conditions they did was an attractive component of the OOPE.

The OOPE provided an opportunity for an accelerated year of learning and development for the doctors that contributed to both their professional and personal development. Many believed their work experience in SA would make them a better doctor for the NHS, and they identified various skills, knowledge and experiences that would be directly relevant to their future work in the NHS. In addition to their general growth and development, their improved ability to work in resource limited settings, a greater appreciation for the NHS and a better understanding of working within foreign health care systems were important gains from their time in SA.

Evaluations of similar programmes such as the International Health Fellowship Program (IHFP) indicate a positive influence on the careers of doctors following their placement abroad. Doctors had demonstrated a strong preference to work with underserved populations and engage in community service activities [18]. Further reviews of this programme reveal that these doctors possess a greater understanding of the challenges of working in areas with scarce resources [19].

These temporary placements support a global call for doctors in developed countries to have a broader knowledge of tropical disease and newly emerging infections, while being culturally sensitive to the increasing number of international travellers and ethnic minority populations [20]. It is argued that this exposure to global health issues can play a role in doctors specialising in primary care medicine and practicing among poor and ethnic communities [20]. Shah points out, however, that these placements have the potential to do more harm than good in these settings when doctors exceed their actual capabilities. In a context where an increasing number of medical students are encountering global health challenges through temporary placements abroad, Shah emphasises the responsibility of medical schools to establish programmes dedicated to global health education. Medical training programmes need to provide adequate and formalised preparation for both the clinical and ethical challenges of working in resource-poor settings [21].

### Limitations

There are several limitations to this study. With one researcher conducting the data analysis, inter-coder reliability could not be established; however other methods for ensuring the trustworthiness of the data were utilised. The study did not include the perspectives of supervisors or fellow colleagues at the hospitals with regard to the structure of the OOPE or the benefits associated with the programme. The majority of the sample were eight months into their OOPE at the time of the study and may not have realised the full benefit of their experience at the time of the interview. Furthermore, views and opinions about whether they would return to SA or future career plans may be context-bound and not endure post departure from SA. With so few of the sample being ‘returnees’ to the UK, the study is limited with regards to its insights on transferability of these benefits and experiences to the NHS.

## Conclusion

This study provides evidence to suggest that North–south partnerships that bring sustainable inflows of foreign HPs into rural health facilities in SA, such as the OOPE run by the London GP Deanery and AHP, have the potential to create substantial benefits for doctors, both in terms of their medical skills and competencies and softer skills. The British trainee GPs gained important skills and competencies during their time in SA that would assist them in fulfilling a number of requirements for their speciality training and, which some believed, would make them better doctors for the UK NHS.

Further research is required to evaluate the extent to which this arrangement is beneficial to the host facilities and whether these trainee GPs are optimally utilised.
